# Digital Twin-Enabled Online Battlefield Learning with Random Finite Sets

**DOI:** 10.1155/2021/5582241

**Published:** 2021-05-13

**Authors:** Peng Wang, Mei Yang, Jiancheng Zhu, Yong Peng, Ge Li

**Affiliations:** College of Systems Engineering, National University of Defense Technology, Changsha 410073, China

## Abstract

The digital twin is becoming the most promising emerging technology in the field of unmanned combat and has the potential to innovate future combat styles. Online battlefield learning is one of the key technologies for supporting the successful application of digital twin in unmanned combat. Since there is an urgent need for effective algorithms for online learning the battlefield states in real time, a new random finite set- (RFS-) based algorithm is proposed in the presence of detection uncertainty including clutters, missed detection, and noises. The system architecture and operational mode for implementing the digital twin-enabled online battlefield learning are provided. The unmanned ground vehicle (UGV) is employed as the experimental subject for systematically describing the proposed algorithm. The system architecture for implementing the digital twin-enabled online battlefield learning is firstly given, and its operational mode is also described in detail. The RFS-based digital twin models including the battlefield state model, UGV motion model, and sensor model are designed. The Bayesian inference is adopted, and the probability hypothesis density (PHD) filter is modified to implement the online learning process. At last, a group of experiments are conducted to verify the performance and effectiveness of the proposed algorithm. The research work in this paper will provide a good demonstration of the application of digital twin in unmanned combat.

## 1. Introduction

The adoption of unmanned vehicles brings both great autonomy and new technical challenges to modern warfare. Unmanned vehicles such as unmanned ground vehicles (UGVs) hold great promise for future combat operations and have already been used in several recent military conflicts in Syria and Afghanistan [1–3]. UGVs are the vehicles that operate while in contact with the ground and without a human presence on board. How to feed back the effective information collected from the real battlefield to the simulation space and how to enable the benefits of future paradigms, such as the Cyber-Physical Systems (CPSs) and digital twin, are big challenges for unmanned combat [4–7]. In this paper, we employ the UGV as the experimental subject to specify our contributions in implementing digital twins in unmanned combat.

Due to the data separation between the real battlefield and its models, it is difficult to achieve the automatic flow of information in a closed loop. Digital twin provides a new and effective way to solve this problem. It can enable the real-time bidirectional interoperability between the real world and virtual simulation space and is also an effective way to enable efficient real-time data sharing throughout the entire operational process including intelligent monitoring, prediction, digital representation, evaluation, decision support, and battlefield learning [8–10].

Battlefield refers to the environment constituted by all the objective factors in the battlespace except the combatants and weapons. All kinds of combat operations are inseparable from the specific battlefield. Battlefield has an important influence on the course and outcomes of combat operations. Combat entities can receive inputs from and provide outputs to the battlefield. The combat intention of the combat entity is realized through its interaction with the battlefield.

Battlefield learning means sensing the entities on the battlefield rapidly, understanding the current situation comprehensively, and predicting future status accurately before decision-making [11]. Battlefield learning is important for predicting future situations and evaluating the operational effectiveness of different actions. Battlefield learning helps to improve the commander's understanding of the situation as a whole and form a basis for decision-making. It is also very important for the commanders' real-time monitoring and perception of the dynamic situation [12].

Based on the classical definition of battlefield learning, online battlefield learning is the process of perceiving an existing battlefield and anticipating how it may evolve in the future. It is useful for obtaining knowledge of the previously unknown battlefield while the real combat process is proceeding simultaneously [13]. Online battlefield learning is also extremely important for generating plans and online decision support for security patrol [14].

In the military simulation, computer-generated force (CGF) is the virtual combat force object which is created by a computer and can control or guide all or part of its action and behavior [15]. The core task of constructing CGF is to model the behavior of combat entities on the battlefield. Online battlefield learning is one of the key technologies of CGF and has a broad application prospect. CGF depends on the online learning battlefield to fuse the data generated by the sensors in the battlefield and generate the real-time battlefield states online.

In recent years, the digital twin has become a hot topic, as well as the representative intelligence in all fields from military to people's livelihood [16–19]. Digital twin emphasizes that the virtual object evolves in real time by receiving data from the physical object, thereby keeping consistent with the physical object throughout its entire simulation cycle [20, 21]. In a broad sense, the digital twin is a system composed of physical objects, simulation models, and the real-time dynamic interaction between them. It requires building the simulation models for real entities and simulating their behaviors [22]. It is regarded as the core link between the real and virtual spaces. With the help of various high-performance sensors and high-speed communication technologies, the digital twin can present and predict the actual situation of physical entities in near real time by integrating the data of physical entities. It enhances the ability of analysis and simulation and controls the physical entities through the virtual-real interactive interfaces and data fusion algorithms [23]. Key to enable digital twin in unmanned combat is understanding the evolving situations in the battlefield accurately and timely.

In this paper, we focus on learning the battlefield states that consist of significant environmental cues and the UGV states. In order to explore how to implement the digital twin-enabled online battlefield learning, we propose a random finite set- (RFS-) based algorithm which can support real-time interaction, as well as the deep integration and mutually beneficial symbiosis between the virtual and real battlefield. It is the necessary foundation for the successful application of the digital twin in unmanned combat. Our main contribution is designing and implementing a new online battlefield learning algorithm by using the RFS-based Bayesian theory and modifying the probability hypothesis density (PHD) filter [24]. The most important value of the proposed algorithm is to break through the data boundary between the real and virtual battlefield and enable the application of digital twin in unmanned combat. This algorithm can eliminate information islands and realize the tight integration and equal interaction of real and virtual battlefield.

The rest of the paper is structured as follows. A literature review on the recent digital twin and random finite set (RFS) is given in Section 2. We present the system architecture and operational mode of the proposed online battlefield learning algorithm in Section 3. The RFS-based battlefield model, UGV motion model, and sensor model are introduced in Section 4. The design and implementation of the learning process are given in Section 5. Experimental results are detailed in Section 6, and conclusions are given in Section 7.

## 2. Related Works

The digital twin has important research and application value in every stage of online battlefield learning. In the design and demonstration stage, the digital twin can help to improve the evaluation capability of system performance by enabling the equal two-way interaction between the simulation system and the real system. Through the semiphysical simulation, digital twin enhances the ability to quickly locate the design defect, optimize system design, and test the practicability of an online battlefield learning algorithm in execution.

In order to apply the digital twin-enabled online battlefield learning in the operation stage, it is important to realize the bidirectional interaction between the simulation space and the real space. Tao gives the five-dimensional structure models of digital twin and presents six application principles [25, 26]. The digital twin is the best way to realize the interactive integration of real space and simulation space and is highly concerned by many academics and enterprises. Its most important breakthrough is that it is not only a mirror image of the physical world but also accepts real-time data from the physical world and in turn acts on the physical world in real time [22, 27]. Digital twin brings new development opportunities to the combat simulation area, because it can allow commanders to have a complete digital footprint of the battlefield from beginning to end [28, 29]. The real-time dynamic interaction between the virtual world and the physical world is the foundation of the digital twin, as well as the main challenge of modeling and simulation. Some researchers present a digital twin-driven manufacturing cyber-physical system for parallel controlling of the smart workshop. By using the decentralized digital twin models, they successfully connect cyberspace and physical space.

Online battlefield learning needs autonomy in the operation stage. A decentralized multiagent system is also a new approach for implementing online battlefield learning, such as blockchain and CGF. Some researchers have discussed how to use blockchain to overcome the cybersecurity barriers for achieving intelligence in Industry 4.0 and introduced eight cybersecurity issues in manufacturing systems. Some researchers have surveyed the ability of blockchain for overcoming the barriers and examined the literature on the manufacturing system perspective and the product lifecycle management perspective. Ali et al. provided a survey of all aspects of multiagent systems, starting from definitions, features, applications, challenges, and communications to evaluation. They also gave a classification on multiagent system applications and challenges along with references for further studies [30].

RFS provides a novel unified probabilistic way for fusing real-time battlefield data [31]. The conventional battlefield learning algorithms usually depend on the vector-based data representation and fail to support the digital twin in real time. The vector-based representation requires the dimension and elements' order in each vector to be equal and fixed. It also needs necessary operations outside of the Bayesian recursion to ensure the consistency of the vectors. The determination of newly observed measurements and missed measurements is implementing through vector augmentation and truncation which are very computationally intensive and irreversible. In this paper, we propose employing the random set theory to overcome these disadvantages. The proposed RFS-based algorithm can overcome the limitations of conventional algorithms very well, because it takes into account a more realistic situation where the randomly varying number of targets and measurements, detection uncertainty, false alarms, and association uncertainty are all taken into consideration.

## 3. System Architecture

**Figure 1:**
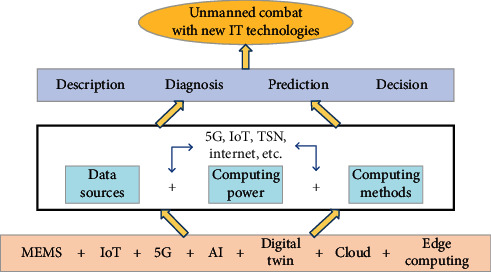
The relationship between new information technologies and unmanned combat.

With the rapid development of emerging information technologies, such as artificial intelligence (AI), cloud computing, edge computing, digital twin, and Internet of Things (IoT), the combat style has also been undergoing profound changes. New information technologies have facilitated the birth, development, and application of unmanned combat. Just as it is shown in Figure 1, new information technologies provide more diverse data sources, more powerful computing power, and more efficient computing methods for the key activities of unmanned combat including description, diagnosis, prediction, and decision.

**Figure 2:**
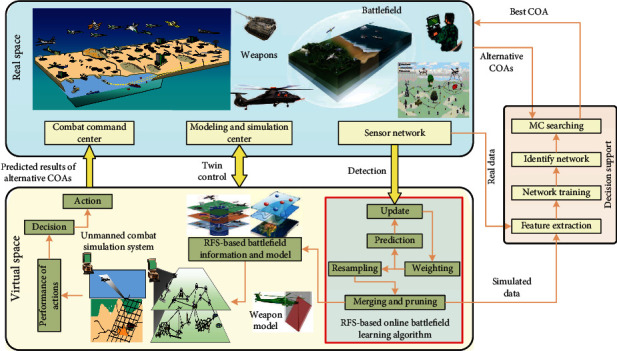
The operational mode of digital twin-enabled online battlefield learning in unmanned combat.

The operational mode of the digital twin-enhanced online battlefield learning consists of five elements, i.e., computing services, physical entities, simulation models, connected data, and the connection between them. As shown in Figure 2, digital twin enables the bidirectional real-time mapping and interaction between real battlefield and its simulation model. Simulation models of the real combat entities are employed to reflect and predict their behaviors in real space. On the other hand, through the RFS-based battlefield states generated by the online battlefield learning algorithm, the combat simulation systems could guide the military commanders to respond to situation changes and choose the optimal courses of action (COA). Digital twin realizes the closed-loop optimization in the entire process from observing, orienting, and deciding to act. The simulation aspect of digital twin means building digital models of weapons, soldiers, or battlefield and executing all the models in an integrated way. The RFS-based simulation models are executed in parallel with the real battlefield and provide useful knowledge to the commanders.

The battlefield considered in this paper consists of all the significant environmental cues and the states of UGVs. Since GPS and topographic map in actual combat are most likely be disabled, location and mapping for unmanned vehicles can only be obtained with the help of the equipped sensors. The RFS-based online battlefield learning algorithm plays a central role in the virtual space. It provides simulated battlefield information to the decision support system to train the deep learning network system. It can also generate real-time battlefield information to the unmanned combat simulation system and helps to evaluate the possible outputs of available COAs.

For combat simulation, the battlefield provides spatial-temporal constraints for all participating actors. The simulated combat objects are deployed and controlled in the virtual space. They learn the battlefield that consists of other combat objects and significant environmental cues by using the proposed algorithm. The combat simulation system in the virtual space is used as a decision-making aid tool that assists the commanders to evaluate all the available COAs. It is in charge of choosing the optimal COA. The proposed online battlefield learning algorithm aims at analyzing and understanding operational activity in the real space at a given time. It can help to make the right decision and predict the future situation. It is the key technology for enabling and implementing digital twin-enabled online battlefield learning in unmanned combat.

**Figure 3:**
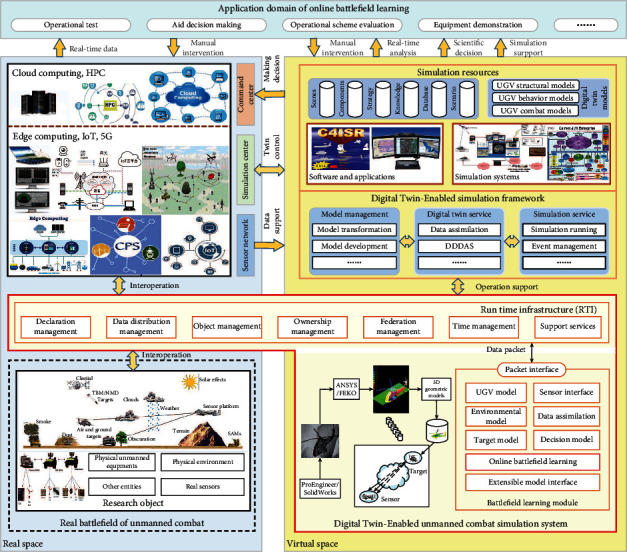
The system architecture of digital twin-enabled unmanned combat simulation.

Corresponding to the operational mode, the system architecture of digital twin-enabled online battlefield learning in unmanned combat is shown in Figure 3. The runtime infrastructure (RTI) is adopted to provide the simulation services to support the interconnection and interoperation for the entities in the real space and the simulation models in the virtual space. This system architecture employs digital twin and RTI to support real-time interaction between the virtual and real battlefield. By this means, it can realize the deep integration and mutually beneficial symbiosis between the virtual and real battlefield. The proposed algorithm can synchronously learn the number and position of the significant environmental cues (or landmarks) in the battlefield that exist in the sensor's field of view (FoV). It also has the advantages of precise mapping, virtual-real interaction, stereoperception, intelligent intervention, and other characteristics.

## 4. RFS-Based Simulation Models

The digital twin-enabled battlefield modeling consists of three aspects. The first one is modeling the battlefield states including cues (or landmarks). The second one is modeling the UGV movement. The third one is modeling the sensors equipped on the UGV. In order to overcome the data association uncertainty problem under high clutters and measurement noises, the RFS-based modeling method is employed to fully integrate data association uncertainty into battlefield learning. The key of the proposed algorithm is to represent the battlefield states by using RFS. The derivation of the simulation models depends on RFS. RFS is the theory proposed by Mahler for implementing RFS in engineering applications [32]. The RFS-based models are the twinning models that are executed in parallel with the real entities and provide new knowledge about the real battlefield [8, 27].

The vector-based representation of the battlefield has been demonstrated to have some mathematical consequences, such as the ordering of significant environmental cues, data association problems, and element management problems. In addition, for the dynamic random scene, how to quantify the errors of the learned results generated by the vector-based Bayesian inference is also a great challenge. The abovementioned problems are usually solved by augmenting or truncating vectors outside of the Bayesian inference process. This will lead to the problem that the Bayesian optimality can only be achieved on the subset of the battlefield that is defined in advance. In this section, we give the RFS-based models which can solve these problems systematically.

The difficulty of RFS-based Bayesian inference is its computational complexity. To solve this problem, Mahler proposed the PHD (probability hypothesis density) filter. The PHD of the posterior probability density *f*_*k|k*_(**X**_*k*_*| ***Z**_*k*_) is denoted by *v*_*k|k*_(**x***| ***Z**_*k*_) and is a density function defined on the single object state **x** ∈ **X**_0_ as follows:

(1) $$\begin{array}{c} v_{k|k}\left(x|Z_{k}\right)=\int f_{k|k}\left(\left\{x\right\}\cup X_{k}|Z_{k}\right)\delta X_{k}. \end{array}$$

Here, **Z**_*k*_ denotes the RFS of detection received at time *k*, and **X**_*k*_ denotes the RFS of states at time *k*. We use the abbreviation *v*_*k|k*_(**x**)=*v*_*k|k*_(**x***| ***Z**_*k*_). In point theory, *v*_*k|k*_(**x**) is defined as the intensity density. It is not a probability density and represents the density of the expected number of points at **x**. Given any subspace **S** of single object state space **X**_0_, the integral ∫_**S**_*v*_*k|k*_(**x**)d**x** is the expected number of objects in **S**.

### 4.1. RFS-Based Battlefield Representation

We adopt the RFS-based battlefield representation; here, *S* denotes the RFS that represents the entire unknown battlefield. In addition, in order to assist in operational decision-making, we also relate the battlefield *S* to the UGV state *X*. RFS *S*_*k*−1_, which is based on the UGV state *X*_0:*k*−1_=[*X*_0_, *X*_1_,…, *X*_*k*−1_] at time *k* − 1, is used to denote the battlefield that has been explored. *S*_*k*−1_ is the RFS of the battlefield states which consists of significant environmental cues and is the intersection of the union of all FoVs and the entire battlefield state. Thus, *S*_*k*−1_ can be represented as follows:

(2) $$\begin{array}{c} S_{k-1}=S\cap \text{FoV}\left(X_{0:k-1}\right). \end{array}$$

Here, FoV(*X*_0:*k*−1_)=FoV(*X*_0_) ∪ FoV(*X*_1_) ∪ ⋯∪FoV(*X*_*k*−1_). FoV depends on the UGV states at time *k* − 1. The learned battlefield at time *k* can be obtained based on *S*_*k*−1_ in the following way:

(3) $$\begin{array}{c} S_{k}=S_{k-1}\cup \left(\text{FoV}\left(X_{k}\right)\cap {\bar{S}}_{k-1}\right), \end{array}$$

where ${\bar{S}}_{k-1}=S-S_{k-1}$ represents the unexplored battlefield namely, the set of significant environmental cues that are not in *S*_*k*−1_. RFS *B*_*k*_(*X*_*k*_) denotes the learned battlefield which has appeared in the FoV for the first time. Therefore, the battlefield transition process can be modeled as

(4) $$\begin{array}{c} f_{S}\left(S_{k}|S_{k-1},X_{k}\right)=\sum_{W\subseteq S_{k}}f_{S}\left(W|S_{k-1}\right)f_{B}\left(S_{k}-W|X_{k}\right), \end{array}$$

where *f*_*S*_(*W|S*_*k*−1_) denotes the state transition density of battlefield from time *k* − 1 to time *k*, and*f*_*B*_(*S*_*k*_ − *W|X*_*k*_) denotes the density of the RFS *B*(*X*_*k*_).

### 4.2. RFS-Based UGV Motion Model

The location of UGV can be represented by the state vector *X*=[*x*, *y*, *θ*]^*T*^. The UGV motion model characterizes the state transition between *X*_*k*−1_=[*x*_*k*−1_, *y*_*k*−1_, *θ*_*k*−1_]^*T*^ and *X*_*k*_=[*x*_*k*_, *y*_*k*_, *θ*_*k*_]^*T*^ after inputting the control command **u**_*k*−1_. In this paper, we adopt the following two-dimensional motion model with translational and rotational displacement:

(5) $$\begin{array}{c} X_{k}={\left[\begin{array}{c} x\\ y\\ \theta \end{array}\right]}_{k}\\ =g\left(X_{k-1},u_{k-1}\right)+\delta \\ =g\left({\left[\begin{array}{c} x\\ y\\ \theta \end{array}\right]}_{k},{\left[\begin{array}{c} \delta x\\ \delta y\\ \delta \theta \end{array}\right]}_{k}\right)+\omega_{k},\;\omega_{k}∼\left(0,Q\right). \end{array}$$

In this paper, the specific mathematical expression of *g* is employed as follows:

(6) $$\begin{array}{c} \left[\begin{array}{c} x_{k}\\ y_{k}\\ \theta_{k} \end{array}\right]=\left[\begin{array}{c} x_{k-1}+\frac{t_{k-1}}{\gamma_{k-1}}\left(\cos\left(\theta_{k-1}+\gamma_{k-1}\right)-\cos\left(\theta_{k-1}\right)\right)\\ y_{k-1}+\frac{t_{k-1}}{\gamma_{k-1}}\left(\sin\left(\theta_{k-1}+\gamma_{k-1}\right)-\sin\left(\theta_{k-1}\right)\right)\\ \theta_{k-1}+\gamma_{k-1} \end{array}\right]+\omega_{k}. \end{array}$$

Here, *ω*_*k*_ is used to represent the uncertainty and noise, and **u**_*k*−1_=[*t*_*k*−1_, *γ*_*k*−1_]^*T*^ is the control command that UGV received at time *k* − 1.

### 4.3. RFS-Based Sensor Model

Given the current UGV state RFS *X*_*k*_ and the battlefield RFS *S*_*k*_, the detection RFS can be described as follows:

(7) $$\begin{array}{c} Z_{k}=\underset{s\in S_{k}}{\cup }D_{k}\left(s,X_{k}\right)\cup C_{k}\left(X_{k}\right). \end{array}$$

Here, *D*_*k*_(*s*, *X*_*k*_) denotes the detection RFS related to the significant environmental cue with state *s*, and *C*_*k*_(*X*_*k*_) denotes the clutters RFS, which is related to the UGV state *X*_*k*_. Due to the uncertainty and randomness in the detection process, the number of elements in *Z*_*k*_ is random and may be different from the number of states in *S*_*k*_.

The detection RFS *D*_*k*_(*s*, *X*_*k*_) generated by battlefield state *s* is modeled by Bernoulli RFS. Therefore, there are two forms of *D*_*k*_(*s*, *X*_*k*_). The first one is *D*_*k*_(*s*, *X*_*k*_)=∅ and the probability is 1 − *p*_*D*_(*s*, *X*_*k*_). The other one is *D*_*k*_(*s*, *X*_*k*_)=*z* and the probability is *p*_*D*_(*s*, *X*_*k*_)*g*_*k*_(*z|s*, *X*_*k*_). *X*_*k*_ denotes the UGV state at time *k*, and *p*_*D*_(*s*, *X*_*k*_) denotes the probability of generating detection from *s*. *g*_*k*_(*z|s*, *X*_*k*_) models the likelihood that *s* generates detection *z*. In this paper, *p*_*D*_(*s*, *X*_*k*_)=*p*_*D*_ if the significant environmental cue exists in the sensor's FoV, and *p*_*D*_(*s*, *X*_*k*_)=0, otherwise.

Depending on *X*_*k*_ and *S*_*k*_, the sensor's likelihood function for generating *Z*_*k*_ is represented as follows:

(8) $$\begin{array}{c} g_{k}\left(Z_{k}|X_{k},S_{k}\right)=\sum_{W\subseteq Z_{k}}g_{D}\left(W|S_{k},X_{k}\right)g_{C}\left(Z_{k}-W\right). \end{array}$$

Here, *g*_*D*_(*W|S*_*k*_, *X*_*k*_) is the likelihood function of generating detection RFS *D*_*k*_ for RFS *S*_*k*_, and *g*_*C*_(*Z*_*k*_ − *W*) is the probability density of the clutter RFS *C*_*k*_.

In this paper, the range and bearing sensor is used. The detection generated by the two-dimensional environmental cues at location *s* can be modeled as follows:

(9) $$\begin{array}{c} z_{k}=\left[\begin{array}{c} r_{k}\\ b_{k} \end{array}\right]\\ =h\left(x_{k},s\right)+e_{k}\\ =\left[\begin{array}{c} \sqrt{{\left(x_{s}-x\right)}^{2}+{\left(y_{s}-y\right)}^{2}}\\ \arctan\left(\frac{y_{s}-y}{x_{s}-x}\right)-\theta \end{array}\right]+e_{k},\;e_{k}∼\left(0,R\right). \end{array}$$

Here, $z_{k}={\left[\begin{array}{cc} r_{k} & b_{k} \end{array}\right]}^{T}$ is the range and bearing detection, $s={\left[\begin{array}{cc} x_{s} & y_{s} \end{array}\right]}^{T}$ is the cue's position, and **e**_*k*_ is the noise with covariance **R**.

## 5. Learning Process and Its Implementation

In this section, we give the basic principles, design, and implementation of the proposed algorithm. The process of the proposed algorithm relies on sequentially propagating the joint posterior probability density of the RFS-based battlefield and the UGV state as detection arrives.

### 5.1. RFS-Based Learning Process

With the RFS-based battlefield modeling, the RFS-based Bayesian inference is used to jointly learn the environmental cues' locations and UGV state at every time step. The battlefield RFS can be characterized as follows:

(10) $$\begin{array}{c} p_{k|k}\left(S=\left\{s^{1},s^{2},\ldots ,s^{{\hat{m}}_{k}}\right\}|Z_{0:k},X_{0:k}\right). \end{array}$$

In this paper, we use *p*_*k|k*−1_(*X*_0:*k*_, *S*_*k*_*|Z*_0:*k*−1_, *U*_0:*k*−1_, *X*_0_) to denote the predicted distribution of the battlefield state and *p*_*k|k*_(*X*_0:*k*_, *S*_*k*_*|Z*_0:*k*_, *U*_0:*k*−1_, *X*_0_) to denote the a posteriori distribution of the battlefield state. The knowledge of the battlefield can be propagated by the following prediction and update process:

(i) Predict the battlefield state by using the previous battlefield states and input parameters:
  (11) $$\begin{array}{c} p_{k|k-1}\left(X_{0:k},S_{k}|Z_{0:k-1},U_{0:k-1},X_{0}\right)\\ =\int f\left(X_{0:k},S_{k}|X_{0:k-1},S_{k-1},U_{k-1}\right)\times p_{k-1|k-1}\left(X_{0:k-1},S_{k-1}|Z_{0:k-1},U_{0:k-2},X_{0}\right)\text{d}X_{k-1}. \end{array}$$
(ii) Update the battlefield state depending on the received detection RFS *Z*_*k*_:
  (12) $$\begin{array}{c} p_{k|k}\left(X_{0:k},S_{k}|Z_{0:k},U_{0:k-1},X_{0}\right)=\frac{g_{k}\left(Z_{k}|S_{k},X_{k}\right)p_{k|k-1}\left(X_{0:k},S_{k}|Z_{0:k-1},U_{0:k-1},X_{0}\right)}{\iint g_{k}\left(Z_{k}|S_{k},X_{k}\right)p_{k|k-1}\left(X_{0:k},S_{k}|Z_{0:k-1},U_{0:k-1},X_{0}\right)\text{d}X_{k}\delta S_{k}}. \end{array}$$

Here, *δ* implies set integration.

**Figure 4:**
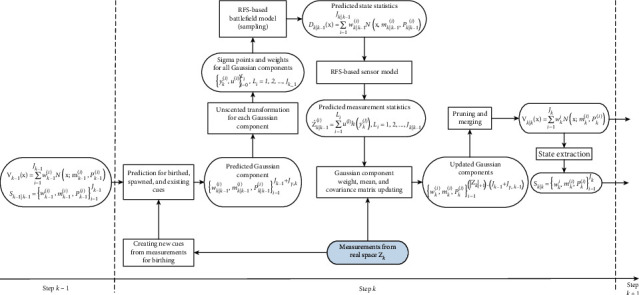
The online battlefield learning process based on Gaussian mixture-based PHD filter.

In this paper, the PHD filter is employed to implement the RFS-based Bayesian recursion [24, 33–35]. We modify and extend the Gaussian mixture-based PHD filter with a particle filter. The Gaussian mixture PHD filter is applied to learn the number and locations of the environmental cues, and the particle filter is applied to learn the UGV state at the same time. The computing process of online battlefield learning by modifying PHD filter is shown in Figure 4. The Bayesian recursion encapsulates the inherent uncertainty of the number of significant environmental cues that may be caused by detection uncertainty, clutters, UGV maneuvers, and the uncertainty related to detection noises.

The main challenge of online battlefield learning is how to learn the number and location of environmental cues while estimating the UGV state at the same time. In this paper, we partition the battlefield state into two kinds: *s* for environmental cues and *X*_*k*_^(*i*)^ for UGV movement. We can analytically integrate out *s* provided that we know *X*_0:*k*_^(*i*)^. This means that even though we only have the sample sets *X*_0:*k*_^(*i*)^, we can also represent *p*(*s|X*_0:*k*_^(*i*)^) successfully. Thus, each particle *X*_*k*_^(*i*)^ represents a value for *s*. The advantage of this approach is that we can reduce the dimensionality of state space in which we are sampling and reduce the error of the learned battlefield.

Here, the Gaussian mixture PHD filter is applied to propagate each PHD that depends on the UGV state. The location of environmental cues in the battlefield is characterized by the Gaussian components of the mixture, and the number of cues in the battlefield is characterized by masses of all the Gaussian components. In this paper, the PHD at time *k* − 1 is characterized by the following *N* particles:

(13) $$\begin{array}{c} {\left\{w_{k-1|k-1}^{\left(i\right)},X_{0:k-1}^{\left(i\right)},v_{k-1|k-1}^{\left(i\right)}\left(s|X_{0:k-1}^{\left(i\right)}\right)\right\}}_{i=1}^{N}, \end{array}$$

where *X*_0:*k*−1_^(*i*)^=[*X*_0_^(*i*)^, *X*_1_^(*i*)^, *X*_2_^(*i*)^,…, *X*_*k*−1_^(*i*)^] is the *i*th hypothesized UGV state set, *w*_*k*−1*|k*−1_^(*i*)^ denotes the weight, and *v*_*k*−1*|k*−1_^(*i*)^(*s|X*_0:*k*−1_^(*i*)^) is the related PHD. The posterior distribution is approximated by the following set of weighted particles:

(14) $$\begin{array}{c} {\left\{w_{k|k}^{\left(i\right)},X_{0:k}^{\left(i\right)},v_{k|k}^{\left(i\right)}\left(s|X_{0:k}^{\left(i\right)}\right)\right\}}_{i=1}^{N}. \end{array}$$

In this paper, *v*_*k*−1*|k*−1_^(*i*)^(*s|X*_*k*−1_^(*i*)^) is the prior PHD of the battlefield states for the *i*th particle related to the *i*th UGV trajectory. *v*_*k*−1*|k*−1_^(*i*)^(*s|X*_*k*−1_^(*i*)^) can be represented by the following Gaussian mixture:

(15) $$\begin{array}{c} v_{k-1|k-1}^{\left(i\right)}\left(s|X_{k-1}^{\left(i\right)}\right)=\sum_{j=1}^{J_{k-1|k-1}^{\left(i\right)}}\eta_{k-1|k-1}^{\left(i,j\right)}N\left(s;\mu_{k-1|k-1}^{\left(i,j\right)},P_{k-1|k-1}^{\left(i,j\right)}\right), \end{array}$$

which consists of *J*_*k*−1*|k*−1_^(*i*)^ Gaussian components. For *j*th Gaussian component, *η*_*k*−1*|k*−1_^(*i*, *j*)^ is predicted weight, *μ*_*k*−1*|k*−1_^(*i*, *j*)^ is mean, and *P*_*k*−1*|k*−1_^(*i*, *j*)^is covariance. The PHD of the new environmental cue for the sampled state *X*_*k*_^(*i*)^ at time *k* is represented by *b*(*s|Z*_*k*−1_, *X*_*k*_^(*i*)^). *b*(*s|Z*_*k*−1_, *X*_*k*_^(*i*)^) is also a Gaussian mixture and can be represented as follows:

(16) $$\begin{array}{c} b\left(s|Z_{k-1},X_{k}^{\left(i\right)}\right)=\sum_{j=1}^{J_{b,k}^{\left(i\right)}}\eta_{b,k}^{\left(i,j\right)}N\left(s;\mu_{b,k}^{\left(i,j\right)},P_{b,k}^{\left(i,j\right)}\right), \end{array}$$

where *J*_*b*,*k*_^(*i*)^ is the number of the Gaussian components of the new PHD at time *k*, and*η*_*b*,*k*_^(*i*, *j*)^, *μ*_*b*,*k*_^(*i*, *j*)^, and*P*_*b*,*k*_^(*i*, *j*)^ are the corresponding Gaussian parameters. The predicted PHD is therefore also a Gaussian mixture and can be represented as follows:

(17) $$\begin{array}{c} v_{k|k-1}\left(s|X_{k}^{\left(i\right)}\right)=\sum_{j=1}^{J_{k|k-1}^{\left(i\right)}}\eta_{k|k-1}^{\left(i,j\right)}N\left(s;\mu_{k|k-1}^{\left(i,j\right)},P_{k|k-1}^{\left(i,j\right)}\right). \end{array}$$

Here, *v*_*k|k*−1_(*s|X*_*k*_^(*i*)^) is composed of *J*_*k|k*−1_^(*i*)^=*J*_*k*−1*|k*−1_^(*i*)^+*J*_*b*,*k*_^(*i*)^ Gaussian components that represent the union of the prior PHD *v*_*k*−1*|k*−1_(*s|X*_*k*−1_^(*i*)^) and the PHD of new environmental cues. Since the detection function can also be represented by a Gaussian mixture, the posterior PHD *v*_*k|k*_(*s|X*_*k*_^(*i*)^) can be represented by a Gaussian mixture as follows:

(18) $$\begin{array}{c} v_{k|k}\left(s|X_{k}^{\left(i\right)}\right)=v_{k|k-1}\left(s|X_{k}^{\left(i\right)}\right)\cdot \left[1-p_{D}\left(s|X_{k}^{\left(i\right)}\right)+\sum_{z\in Z_{k}}\sum_{j=1}^{J_{k|k-1}^{\left(i\right)}}v_{G,k}^{\left(i,j\right)}\left(z,s|X_{k}^{\left(i\right)}\right)\right]. \end{array}$$

The components of equation (18) are given as follows:

(19) $$\begin{array}{c} v_{G,k}^{\left(i,j\right)}\left(z,s|X_{k}^{\left(i\right)}\right)=\eta_{k|k}^{\left(i,j\right)}\left(z|X_{k}^{\left(i\right)}\right)N\left(s;\mu_{k|k}^{\left(i,j\right)},P_{k|k}^{\left(i,j\right)}\right),\\ \eta_{k|k}^{\left(i,j\right)}\left(z|X_{k}^{\left(i\right)}\right)=\frac{p_{D}\left(s|X_{k}^{\left(i\right)}\right)\eta_{k|k-1}^{\left(i,j\right)}q^{\left(i,j\right)}\left(z,X_{k}^{\left(i\right)}\right)}{c\left(z\right)+\sum_{l=1}^{J_{k|k-1}^{\left(i\right)}}p_{D}\left(s|X_{k}^{\left(i\right)}\right)\eta_{k|k-1}^{\left(i,l\right)}q^{\left(i,l\right)}\left(z,X_{k}^{\left(i\right)}\right)}. \end{array}$$

Here, *q*^(*i*, *j*)^(*z*, *X*_*k*_^(*i*)^)=*N*(*z*; *H*_*k*_*μ*_*k|k*_^(*i*, *j*)^, *S*_*k|k*_^(*i*, *j*)^). The terms *μ*_*k|k*_, *P*_*k|k*_, and *S*_*k|k*_ can be got through standard Kalman filters; here, we adopt the unscented Kalman filter [36].

We assume that the number of clutters in *C*_*k*_ complies with the Poisson distribution, and the elements comply with uniform distribution over the battlefield state space. Then, the clutter PHD can be represented by *c*(*z*)=*λ*_*c*_*U*(*z*); here, *λ*_*c*_ denotes the averaged number of clutters, and *U*(*z*) complies with a uniform distribution. In order to reduce the amount of calculation, we use pruning and merging methods to reduce the number of Gaussian components of the updated distribution [37].

The posterior UGV state *p*_*k*_(*X*_1:*k*_) is sampled by ${\left\{{\tilde{w}}_{k|k}^{\left(i\right)},{\tilde{X}}_{k}^{\left(i\right)}\right\}}_{i=1}^{N}$ with

(20) $$\begin{array}{c} {\tilde{X}}_{k}^{\left(i\right)}∼f\left({\tilde{X}}_{k}^{\left(i\right)}|X_{k-1}^{\left(i\right)},U_{k-1}\right),\\ {\tilde{w}}_{k}^{\left(i\right)}=\frac{g_{k}\left(Z_{k}|Z_{0:k-1},{\tilde{X}}_{0:k}^{\left(i\right)}\right)f\left({\tilde{X}}_{k}^{\left(i\right)}|X_{k-1}^{\left(i\right)},U_{k-1}\right)}{f\left({\tilde{X}}_{k}^{\left(i\right)}|X_{0:k-1}^{\left(i\right)},U_{k-1}\right)}w_{k-1}^{\left(i\right)}. \end{array}$$

The weights should be normalized as $\sum_{i=1}^{N}{\tilde{w}}_{k}^{\left(i\right)}=1$. With the resampling step [24], we can get the resampled particles ${\left\{{\tilde{w}}_{k}^{\left(i\right)},{\tilde{X}}_{k}^{\left(i\right)}\right\}}_{i=1}^{N}$. By choosing the UGV transition density as the proposal density, we get the weight as follows:

(21) $$\begin{array}{c} {\tilde{w}}_{k}^{\left(i\right)}=g_{k}\left(Z_{k}|Z_{0:k-1},{\tilde{X}}_{0:k}^{\left(i\right)}\right)w_{k-1}^{\left(i\right)}. \end{array}$$

By assuming that there is only one environmental cue $\bar{s}$ in the battlefield, then, we can get

(22) $$\begin{array}{c} g_{k}\left(Z_{k}|Z_{0:k-1},X_{0:k}\right)\approx \frac{1}{\Gamma }\left[\left(\left(1-p_{D}\left(\bar{s}|X_{k}\right)\right)\kappa_{k}^{Z_{k}}+p_{D}\left(\bar{s}|X_{k}\right)\sum_{z\in Z_{k}}\kappa_{k}^{Z_{k}-\left\{z\right\}}g_{k}\left(z|\bar{s},X_{k}\right)\right)v_{k|k-1}\left(\bar{s}|X_{0:k}\right)\right], \end{array}$$

with

(23) $$\begin{array}{c} \Gamma =\exp\left({\hat{m}}_{k|k-1}-{\hat{m}}_{k|k}+\int c_{k}\left(z\right)\text{d}z\right)v_{k|k}\left(\bar{s}|X_{0:k}\right). \end{array}$$

Here, ${\hat{m}}_{k|k-1}=\sum_{j=1}^{J_{k|k-1}^{\left(i\right)}}\eta_{k|k-1}^{\left(i,j\right)}$ and${\hat{m}}_{k|k}=\sum_{j=1}^{J_{k|k}^{\left(i\right)}}\eta_{k|k}^{\left(i,j\right)}$.

### 5.2. Implementation

**Figure 5:**
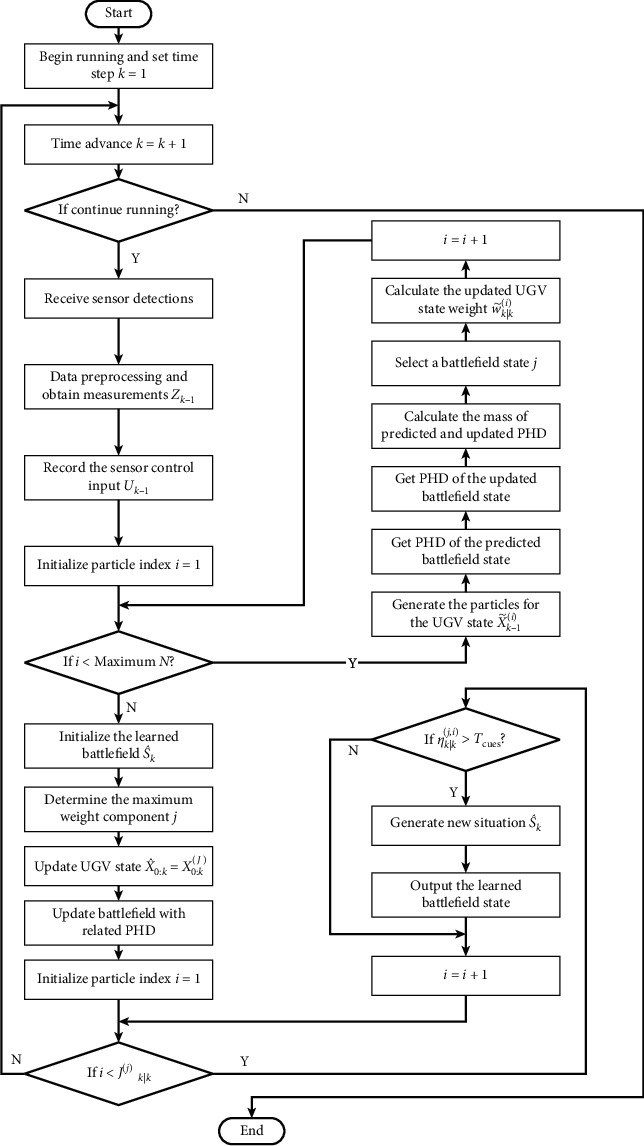
The flow diagram of the RFS-based online battlefield learning algorithm.

According to the learning process given above, we give the concrete realization method of the proposed algorithm in this section. We use C++ to write the experimental program for this algorithm. The C++ library dependencies such as Eigen (version 3.0.0), Boost (version 1.5.3), and gtest are also used. In order to detail the implementation of the proposed algorithm, the flow diagram of the proposed algorithm is presented in Figure 5. The concrete steps are described in Algorithm 1.

The computational complexity of the proposed algorithm is Ο(*m*_*k*_ · |*Z*_*k*_| · *N*) and is linear in the number of landmarks (in the FoV), as well as the number of detections and number of particles for the UGV state.

## 6. Experiments

In this section, a group of experiments are conducted to quantitatively verify the effectiveness and analyze the performance of the proposed algorithm. The virtual machine we used to run our experiments has 4 G of RAM and 6 3.40 GHz Intel CPUs and runs on Unbuntu 14.04 OS. The experimental data used to support the findings of this study are included within the article. The parameters used in this experiment are given in Section 6.1, and the models used in this experiment are given in Section 4.

### 6.1. Experimental Setup

**Figure 6:**
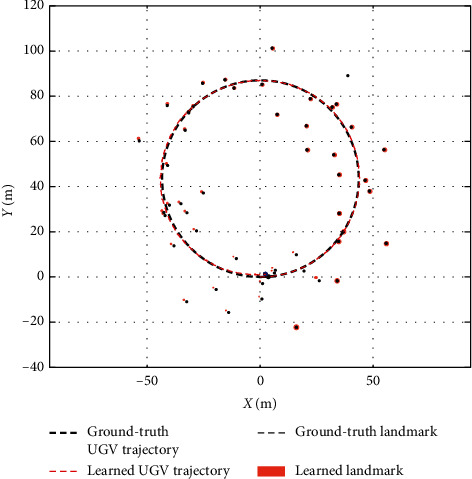
The real and learned battlefield states.

As shown in Figure 6, the UGV patrols in a simulated two-dimensional space. The known ground truth (including the UGV states and locations of landmarks) is generated by the simulation models. The black dots represent the real locations of landmarks, and the black dashed line represents the real UGV states. The number of clutters complies with the Poisson distribution, and the clutter PHD is uniformly distributed.  Table 1 shows some important parameters for the simulation models to generate the ground truth.

The sensor used in this experiment is the range-bearing sensor that can detect landmarks with distances of 5 m to 30 m in any direction. The range measurement standard deviation (std) is 1 m and the bearing measurement std is 2 deg. The maximum FoV of the sensor used by the UGV is 10 m and 360 deg.

### 6.2. Results and Analysis

The experimental results are shown in Figure 6, the red dashed line represents the learned UGV states, and the red points represent the learned locations of landmarks. The collection of red dashed lines and points represents the battlefield states. The results successfully confirm that the proposed algorithm can learn the battlefield states by using sensor detection at runtime.

**Figure 7:**
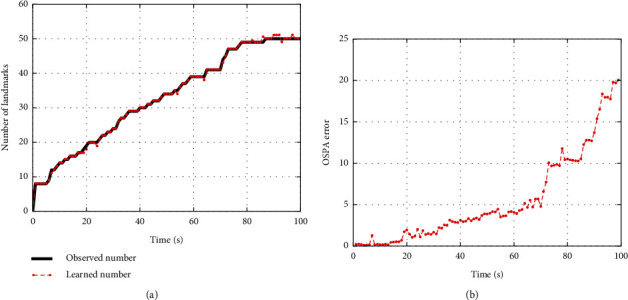
The errors of the learned number and locations of landmarks. (a) The learned and observed number of landmarks. (b) The OSPA errors of the learned landmarks.

**Figure 8:**
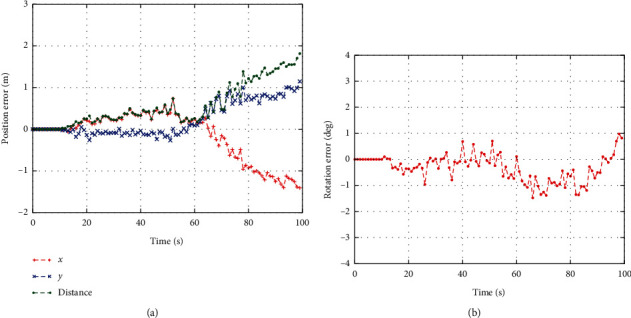
The errors of the learned UGV states. (a) Euclidean errors of the learned UGV states. (b) Orientation errors of the learned UGV states.

In order to quantitatively evaluate the performance of the proposed algorithm, we give the errors of the learned battlefield states in Figures 7 and 8. Figures 7(a) and 7(b) give the errors of the learned number and locations of landmarks. The errors of locations are represented by optimal subpattern assignment (OSPA) distance [38]. We can find out that the performance of the proposed algorithm can satisfy the requirements of simulation and evaluation in unmanned combat.

Consider two sets **X**={**x**_1_,…, **x**_*m*_} and **Y**={**y**_1_,…, **y**_*n*_}, where *m*, *n* ∈ *ℕ*_0_={0,1,…}. Vectors **x** ∈ **X** and **y** ∈ **Y** are taking values from the battlefield state space. The OSPA metric is defined as a distance between sets **X** and **Y**. The OSPA distance of order 1 ≤ *p* ≤ *∞*, with the cut-off parameter *c*, is defined for *m* ≤ *n* as follows:

(24) $$\begin{array}{c} d_{p,c}\left(X,Y\right)={\left[\frac{1}{n}\left(\underset{\pi \in \Pi_{n}}{\min}\sum_{i=1}^{m}{\left(d_{c}\left(x_{i},y_{\pi \left(i\right)}\right)\right)}^{p}+\left(n-m\right)\cdot c^{p}\right)\right]}^{\left(1/p\right)}, \end{array}$$

where Π_*n*_ represents the permutations set of length *m* with elements taken from {1,2,…, *n*}.

The errors of learned UGV states are shown in Figures 8(a) and 8(b). We can find out that the proposed algorithm can generate the learned UGV states with acceptable accuracy. But the errors are increased as time advance. This is caused by the cumulative errors of the UGV states.

**Figure 9:**
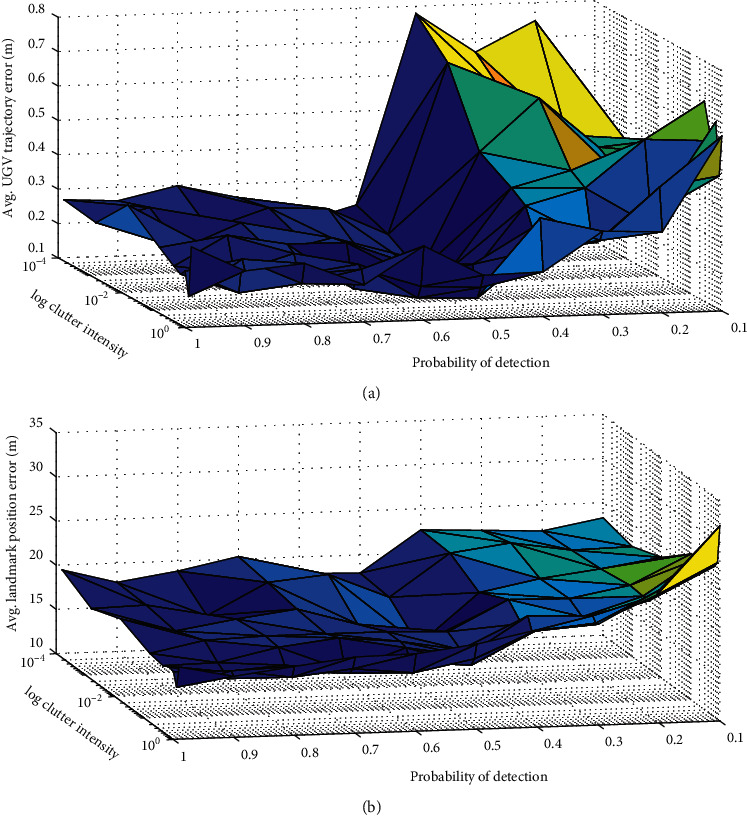
The performance for varying values of probability of detection and clutter intensity. (a) Averaged errors for the learned UGV states. (b) Averaged COLA errors for learned landmarks.

In order to analyze how detection parameters affect the proposed algorithm, the averaged errors of the UGV states and landmarks are generated with different probabilities of detection *p*_*D*_ from 0.1 to 0.99 and clutter intensity *λ*_*c*_ from 0.0001 to 1. For each pair of parameters, 10 simulation runs were carried out. Here, cardinalized optimal linear assignment (COLA) is used to evaluate the errors for the learned landmarks. From Figures 9(a) and 9(b), we can find out that the errors of the learned UGV states increase as *p*_*D*_ decreases. The errors of the learned landmarks only increase slightly as *P*_*D*_ decreases. The increase of *λ*_*c*_ will increase the errors of the learned locations of landmarks, but the effect on the errors of learned UGV states is quite small.

**Figure 10:**
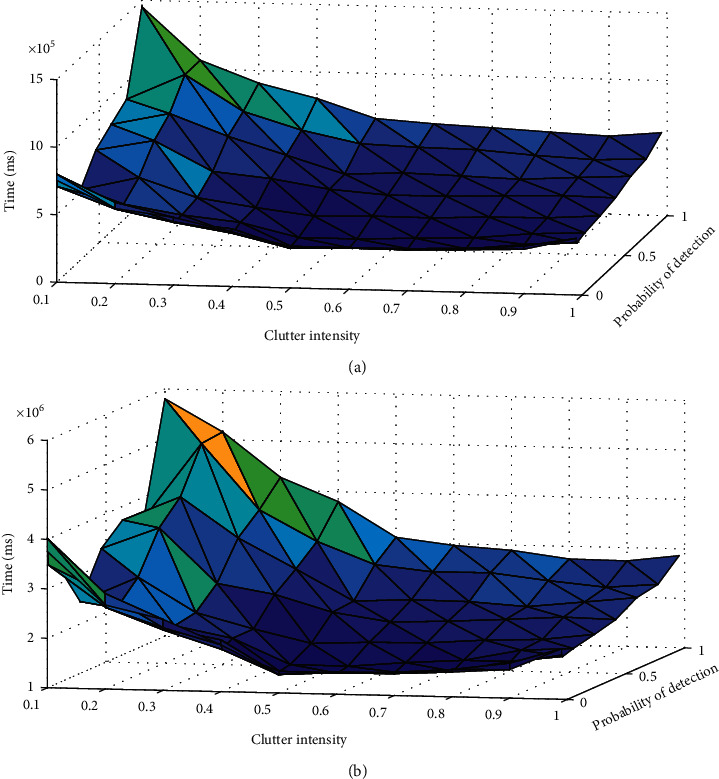
Time cost for varying values of detection probability and clutter intensity. (a) Averaged time cost of the algorithm. (b) Averaged time cost of CPU while the algorithm running.

In order to apply the proposed algorithm in real unmanned combat applications, the time cost should be fully evaluated. As shown in Figure 10, we record 10 simulation runs for each pair of detection probability and clutter intensity, and each simulation run consists of 1000 time steps. The averaged time costs of the proposed algorithm and CPU are shown in Figures 10(a) and 10(b). We can find that the increase of detection probability will increase the time cost, and the decrease of clutter intensity will also increase the time cost. The average time cost for each time step is about 500 ms, and it can satisfy many unmanned combat applications very well.

## 7. Conclusions

Digital twin technology enables real-time dynamic interaction between the real battlefield and the simulation system. Our main contribution is proposing a new online battlefield learning algorithm based on RFS to enable the application of the digital twin in unmanned combat. The digital twin has a broad application prospect in unmanned combat and greatly promotes the innovation of unmanned combat mode. Since the implementation of the digital twin in unmanned combat depends on battlefield understanding, an effective battlefield learning algorithm is quite important. By adopting the RFS-based representation of the battlefield, the proposed algorithm can overcome the limitations of the traditional vector-based representation. The performance of the proposed algorithm is verified by using two groups of experiments. This paper is the first attempt for applying the digital twin to the unmanned combat area and has practical significance for implementing the digital twin in many other areas.

## Figures and Tables

**Algorithm 1:**
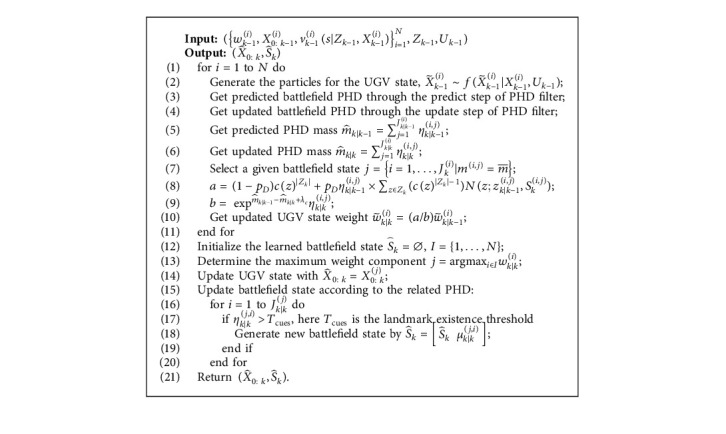
RFS-based online battlefield learning algorithm.

**Table 1:** Partial experimental parameters.

Parameter	Velocity input std. (m/s)	Steering input std. (deg)	Detection probability *p*_*D*_	Clutter rate *λ*_*c*_	Particle number *N*	Landmark existence threshold *T*_cue_
Value	2	2	0.90	0.0001	100	0.5

## Data Availability

The experimental data used to support the findings of this study are included within the article.

## Abbreviations

CGF: Computer-generated force
COA: Courses of action
COLA: Cardinalized optimal linear assignment
EKF: Extended Kalman filter
FoV: Field of view
IoT: Internet of things
OSPA: Optimal subpattern assignment
PHD: Probability hypothesis density
RFS: Random finite set
UGV: Unmanned ground vehicle.
